# Boosting Visible-Light-Driven Hydrogen Evolution Enabled by Iodine-Linked Magnetically Curved Graphene with Mobius-like Electronic Paths

**DOI:** 10.3390/molecules30061302

**Published:** 2025-03-13

**Authors:** Liangjun Cai, Hongxia Liu, Xiaoxiao Yan

**Affiliations:** 1 Jiangxi Province Key Laboratory of Environmental Pollution Prevention and Control in Mining and Metallurgy, Jiangxi University of Science and Technology, Ganzhou 341000, China; cailiangjun2002@163.com; 2 Key Laboratory for Green Chemistry of Jiangxi Province, Jiangxi Normal University, Nanchang 330022, China

**Keywords:** crumpled graphene oxide, spin polarization, heavy-atom effect of group I, photocatalytic hydrogen production, Möbius-like cyclic electron transport pathways

## Abstract

Materials with high electron transfer performance remain a key focus in photocatalytic research, as they can effectively promote the separation of photogenerated carriers and enhance the utilization efficiency of photogenerated electrons. To enhance the effective utilization of photogenerated electrons, the MSIG material was prepared by incorporating the iodine clusters and magnetic Fe_3_O_4_ into the as-synthesized crumpled graphene oxide (CGO) to construct Möbius-like electronic transmission pathways. The introduction of magnetic groups optimized the spin orientation of electrons, facilitating directional electron transport and thereby enhancing the photocatalytic efficiency of the material. Experimental results reveal that, in visible light-driven hydrogen production reactions, the eosin Y (EY)-sensitized Pt-Fe_3_O_4_-MSIG catalyst exhibits outstanding catalytic performance, with a hydrogen production rate of 1.48 mL/h, which is 15 times higher than that of the Pt-Fe_3_O_4_ catalyst. Photoelectrochemical analyses show a significant increase in the catalyst’s fluorescence lifetime, attributed to the Möbius strip-like electron transport channels within the material. Theoretical calculations further support this by demonstrating that the bandgap widening of the CGO reduces the recombination probability of photogenerated carriers, thereby improving their average lifetime. This study offers a novel approach for the design of visible-light-driven photocatalytic materials.

## 1. Introduction

Photocatalytic hydrogen evolution relies on utilizing photogenerated electrons to reduce H⁺ and produce hydrogen [1,2]. However, the recombination of photogenerated electrons and holes is a competitive process that reduces the efficiency of photocatalytic hydrogen production [3,4]. Therefore, inhibiting the recombination of photogenerated electrons and enhancing the effective migration rate of charge carriers play a crucial role in photocatalytic reactions [5]. To improve the separation efficiency of photogenerated carriers, significant efforts have been made, including modulation of the band structure of photocatalysts [6], enhancement of catalyst conductivity [7], design of suitable transport channels for carriers [8], and use of co-catalysts [9].

Graphene, as a promising material, has attracted much attention due to its large theoretical specific surface area (2600 m^2^·g^−1^), high intrinsic electron mobility (200,000 cm^2^·V^−1^·S^−1^), high transmittance (~97.7%), and good electrical conductivity [10,11,12]. However, the hydrophobic nature of graphene flakes makes it more difficult to use in liquid environments. The preparation of graphene oxide (GO) by chemical oxidation, and subsequently reduced graphene oxide (RGO) obtained by chemical reduction, is considered one of the most effective methods to improve graphene as a photocatalytic carrier. The oxygen-containing functional groups on the surfaces of GO and RGO allow them to be dispersed in polar or non-polar solvents and trap reactants more easily. To improve the overall efficiency of the photolytic water reaction, researchers usually add appropriate co-catalysts, such as Pt, Ru and other noble metals. They have larger work functions and lower Fermi energy levels and are prone to form Schottky barriers with semiconductors and participate in hydrogen evolution reactions as excellent electron trapping traps. The addition of photosensitizers such as eosin Y (EY) to the reaction system is also an effective means to improve the activity of photodissociation of water. Min et al. [13] utilized RGO-loaded Pt catalysts for the reduction of aqueous hydrogen in the EY system, and the highest rate of H_2_ production after optimizing the conditions was up to 14.14 μmol·h^−1^. When EY molecules are adsorbed on RGO and excited by visible light, a large energy band shift will form between the EY* and RGO, and the excited electrons will be transferred to the RGO lamellae and reduce H^+^ with the help of Pt. At the same time, the photosensitizer has a wider range of light absorption, which improves the utilization of the system for sunlight. In addition, the oxidation level of GO can be controlled to modulate the band gap to a suitable position. Teng et al. [14] reported that graphite oxide semiconductors with a band gap of 2.4–4.3 eV can stably produce H_2_ from pure water or methanol solution under visible light drive by itself, without the aid of any co-catalyst.

Despite such excellent performance and tunable properties of GO, the high complexation rate of carriers in the catalyst is still one of the main factors hindering the increase of reactivity. Simultaneously constructing carrier transport channels to promote the directional transport of carriers is the most effective method to address the challenge of photogenerated carrier separation [15]. Iodine doping improves electrical conductivity of polymer materials [16,17]. Kalita et al. [18] have demonstrated that electron transfer between atomic iodine and graphene surfaces is enhanced with the help of polyiodides (I_3_^−^ and I_5_^−^), opening new channels for electron transfer on iodine-doped graphene surfaces. In addition to improving the electrical conductivity of graphene, external or local magnetic fields can be applied to modulate the carrier transport behavior [19,20]. In the case of magneto-electric coupling in Co-doped graphene sheets, for example, the spin direction of carriers can be reversed via electric field induction, leading to a spin switching effect [21]. The magnetic field effect in π-conjugated organic semiconductors, as discovered by J. Kalinowski et al. [22], has garnered increasing attention. When a magnetic field is applied parallel to the light-emitting layer, the luminescence intensity, current, and quantum efficiency of the device are altered, with luminescence intensity changing by up to 5% and current variation reaching 2.5%. Zhai et al. [23] considered the internal local equivalent magnetic field generated by the material’s hyperfine interaction and spin-orbit coupling. These studies collectively demonstrate that, under the influence of both external magnetic fields and internal local magnetic fields, electron migration and transport exhibit unique patterns, with time-varying spin angular momentum, spin reversal, and changes in the average spin angular momentum as a function of the magnetic field [24]. In the field of photocatalysis, photocatalysts constructed with magnetic nanomaterials as matrix elements have demonstrated special performance advantages in the research directions of photocatalytic degradation of organic pollutants and solar photocatalytic hydrogen production [25,26,27].

**Figure 1:**
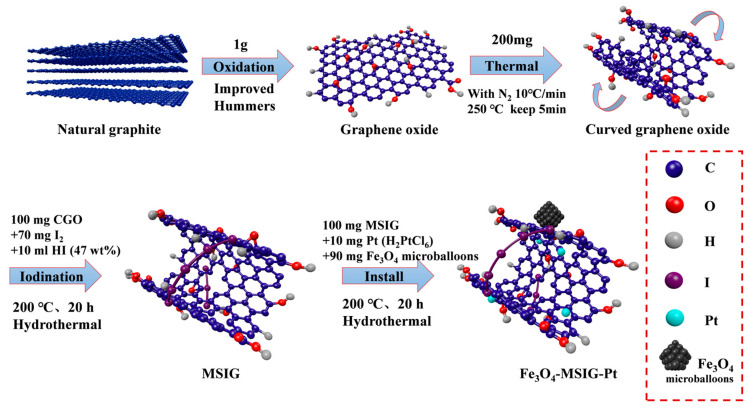
Pt/Fe_3_O_4_-MSIG catalyst preparation mechanism diagram.

In this study, we incorporated magnetic Fe_3_O_4_ units to prepare magnetic iodine-linked cyclic sponge graphene (MSIG-Fe_3_O_4_) (Figure 1). After loading Pt, the material was used as a catalyst for hydrogen evolution under visible light. Our results show that the photocatalytic activity of the Pt/Fe_3_O_4_-MSIG catalyst is significantly improved compared to traditional graphene structures, with a hydrogen production rate up to 1.48 mL/h. Photoelectrochemical characterization revealed that heat treatment increased the number of defects on the catalyst surface, leading to a decrease in conductivity. However, the combined effects of the built-in magnetic field and cyclic electron transport medium substantially enhanced carrier separation efficiency, significantly improving the fluorescence lifetime of the catalyst. We further investigated the electron transport properties of the catalyst using theoretical calculations, which indicated that the bandgap widening in CGO reduces the likelihood of photogenerated carrier recombination, thereby improving their average lifetime.

## 2. Results and Discussion

### 2.1. The XRD and Raman Analysis

**Figure 2:**
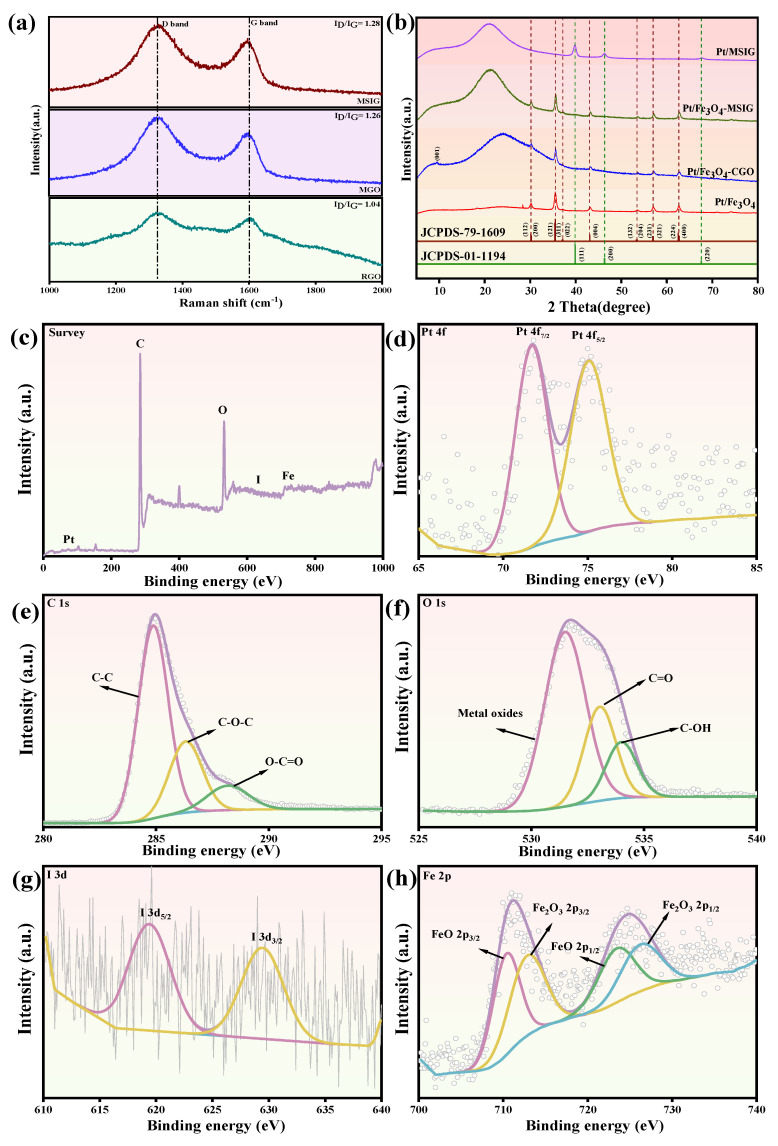
(**a**) The Raman spectrum of MSIG, MGO and RGO materials; (**b**) The XRD spectrum of Pt/MSIG, Pt/Fe_3_O_4_-MSIG, Pt/Fe_3_O_4_-MSIG and Pt/Fe_3_O_4_ catalyst. XPS spectra of the Pt/Fe_3_O_4_-MSIG catalyst. (**c**) The survey XPS spectra of Pt/Fe_3_O_4_-MSIG catalyst; (**d**) Pt 4f; (**e**) C 1s; (**f**) O 1s; (**g**) I 3d; (**h**) Fe 3p.

Raman spectroscopy serves as a potent technique for identifying graphene materials and analyzing the augmentation of surface defect states in carbon materials [28]. To investigate the structural and compositional differences among several catalysts, we conducted the Raman analysis of MSIG, MGO and RGO materials. As depicted in Figure 2a, pristine GO exhibits two distinct peaks: the D band (1346.2 cm^−1^) and the G band (1586.7 cm^−1^), which align with the characteristic peaks of grapheme [29]. However, the D/G intensity ratios (I_D_/I_G_) of MSIG, MGO and RGO are calculated to be 1.28, 1.26, and 1.04, respectively, exceeding that of bare GO, which is 0.686 [30]. This elevation suggests that upon high-temperature heat treatment, two reactions occur vigorously at oxygen-rich regions, releasing significant amounts of H_2_O, CO, and CO_2_ gases, effectively exfoliating stacked GO sheets and leading to the formation of more defects [31]. Moreover, during the high-temperature treatment process, graphene oxide tends to curve to maintain a lower surface energy.

The XRD analysis of Pt/MSIG, Pt/Fe_3_O_4_-MSIG, Pt/Fe_3_O_4_-MSIG and Pt/Fe_3_O_4_ catalysts within the 2θ range of 5 to 80° were shown in Figure 2b. In the spectrum of Pt/Fe_3_O_4_-CGO, the little diffraction peak at 11.91° corresponds to the (001) plane of rGO [32] indicating a highly organized crystal structure with an interlayer spacing of 0.742 nm. This spacing is attributed to the introduction of oxygen-containing functional groups into the crystal lattice during the oxidation of graphite [33]. Another peak at 2θ = 22.95°, corresponding to a d-spacing of 0.386 nm, suggests a lower degree of crystallization and the presence of defects [34]. Compared to the spectrum of Pt/Fe_3_O_4_-MSIG, the diffraction peak at 11.91° completely disappears in Pt/Fe_3_O_4_-CGO, while the peak at 22.95° indicates the existence of defects. We attribute this phenomenon primarily to the exfoliation of graphene oxide and the proliferation of defect sites at elevated temperatures. Peaks located at 35.5°, 43.2°, 53.6°, 57.1°, and 62.7° corresponding to the (200), (121), (004), (321), and (400) planet of Fe_3_O_4_ (JCPDS-79-1609), peaks located at 39.9° and 46.4° corresponding to the (111) and (200) planet of Pt (JCPDS-01-1194). By comparing the spectra of the catalysts, it is found that, in the presence of Fe_3_O_4_, the diffraction peak of Pt is not obvious. This may be due to the good dispersion and lower crystallinity of Pt nano-particles, as can be seen in the catalyst absence of Fe_3_O_4_, diffraction peaks of Pt (111) and Pt (100) crystal appear. The reduction of Pt particles in the catalytic system is also related to the conductivity of the catalyst to a certain extent, which further indicates that the presence of Fe_3_O_4_ can affect the electron transfer performance in the system [35].

### 2.2. XPS Analysis

As shown in Figure 2c–h, a detailed analysis of the X-ray photoelectron spectroscopy (XPS) survey spectra of Pt/Fe_3_O_4_-MSIG was performed to determine the elemental composition of the sample’s surface. Figure 2c displays the full survey spectrum, covering a binding energy range from 0 to 1000 eV, revealing that the Pt/Fe_3_O_4_-MSIG catalyst contains five elements: C, O, I, Fe, and Pt. The peaks corresponding to Pt and I exhibit relatively low intensities, indicating their low concentrations. Figure 2d focuses on the chemical states of Pt, where two distinct peaks at 70.98 eV and 74.48 eV correspond to the Pt 4f_7/2_ and Pt 4f_5/2_ states of metallic Pt. [36]. Figure 2e shows the deconvoluted C 1s spectra, which reveal the presence of various functional groups. Specifically, the peak at 284.6–284.8 eV is attributed to carbon in graphene, while the peaks at 286.9–287.3 eV and 288.2–288.3 eV correspond to C-O-C and O-C=O groups, respectively. The XPS spectrum of O 1s, shown in Figure 2f, displays two peaks at 531.2 eV and 534 eV, which are associated with oxygen-containing functional groups (C-O and C=O) on the material’s surface [37]. Figure 2g presents the XPS spectrum of I 3d, showing peaks at 618.60 eV and 629.96 eV, corresponding to the I 3d_5/2_ and I 3d_3/2_ states [38]. The relatively low intensity of the I 3d peaks suggests a low iodine doping level. Finally, Figure 2h depicts the XPS spectra of Fe 2p, where the peaks at 711.6 eV and 726.6 eV correspond to the Fe 2p_3/2_ and Fe 2p_1/2_ states of Fe_3_O_4_, while additional peaks at 709.8 eV and 723.7 eV are attributed to the Fe 2p_3/2_ and Fe 2p_1/2_ states of FeO [39].

### 2.3. TEM Analysis

**Figure 3:**
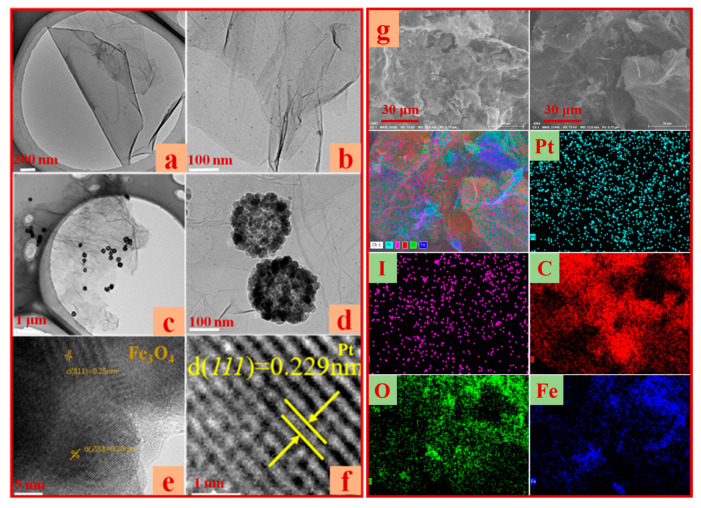
The TEM, HRTEM, and SEM images of Pt/Fe_3_O_4_-MSIG catalysts; (**a**,**b**) TEM of as prepared sponge graphene; (**c**) TEM image of Pt/Fe_3_O_4_-MSIG catalyst; (**d**) TEM image of Fe_3_O_4_; (**e**) HRTEM images of Fe_3_O_4_; (**f**) HRTEM images of Pt; (**g**) Element mappings (EDX analysis) of Pt/Fe_3_O_4_-MSIG catalyst.

The microstructure and morphology of the Pt/Fe_3_O_4_-MSIG catalyst were characterized using transmission electron microscopy (TEM) and scanning electron microscopy (SEM). As depicted in Figure 3a,b, the sponge-like graphene exhibits a wrinkled structure, which can be attributed to the bending of the graphene sheets during high-temperature treatment, resulting in a reduction of surface energy. TEM images of Fe_3_O_4_ microspheres within the Pt/Fe_3_O_4_-MSIG catalyst (Figure 3c,d) reveal that the Fe_3_O_4_ microspheres have an approximate diameter of 200 nm and are uniformly distributed on the MSIG surface. Figure 3e,f present high-resolution TEM (HRTEM) images of Pt/Fe_3_O_4_-MSIG. The interplanar spacings of 0.30 nm and 0.25 nm correspond to the (220) and (311) crystal planes of Fe_3_O_4_, while the 0.229 nm spacing is attributed to the (111) crystal plane of Pt, which aligns with the XRD characterization results [40]. As shown in Figure 3g, the elemental mapping of the Pt/Fe_3_O_4_-MSIG catalyst indicates that the elements C, O, Pt, and Fe are uniformly distributed on the catalyst surface.

### 2.4. UV-Vis Analysis

**Figure 4:**
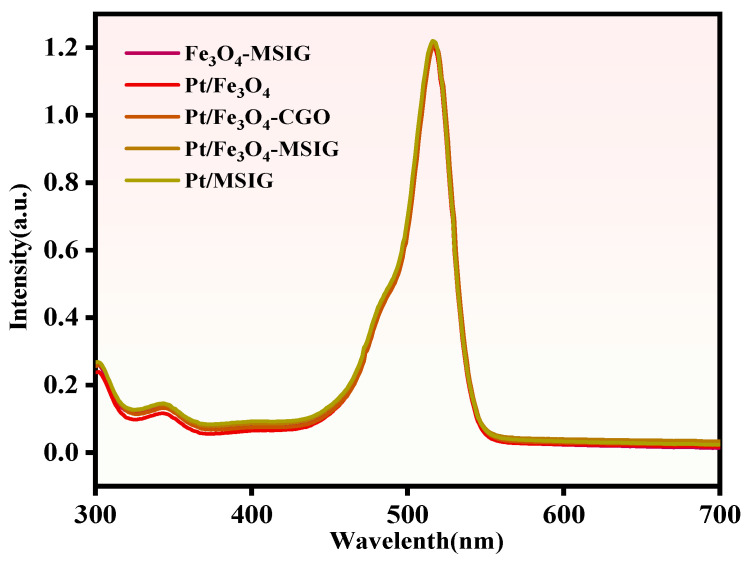
UV–vis absorption spectra of EY sensitized Fe_3_O_4_-MSIG, Pt/Fe_3_O_4_, Pt/Fe_3_O_4_-CGO, Pt/Fe_3_O_4_-MSIG, and Pt/MSIG catalysts.

In photocatalytic reactions, the ability of a catalyst to effectively utilize visible light is of critical importance. As shown in Figure 4, the UV–Vis spectra indicate that upon sensitization with EY (Eosin Y), the Fe_3_O_4_-MSIG, Pt/Fe_3_O_4_, Pt/Fe_3_O_4_-CGO, Pt/Fe_3_O_4_-MSIG, and Pt/Fe_3_O_4_-MSIG catalysts exhibit significant absorption centered around 517 nm, which corresponds to the characteristic absorption of the EY dye molecule. These results highlight the ability of the sensitized Fe_3_O_4_, Pt/Fe_3_O_4_, Pt/Fe_3_O_4_-CGO, Pt/Fe_3_O_4_-MSIG, and Pt/Fe_3_O_4_-MSIG catalysts to absorb visible light with wavelengths greater than 420 nm [41].

### 2.5. Catalytic Activity Performance

**Figure 5:**
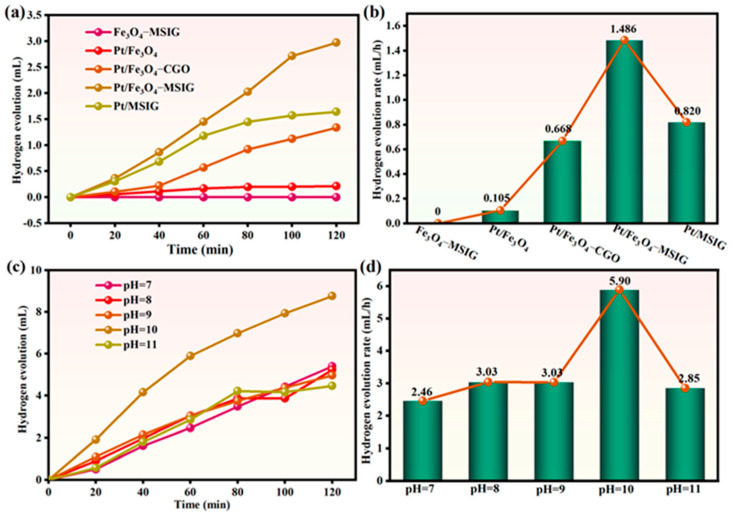
(**a**) Photocatalytic activities for hydrogen evolution over different catalysts under visible light irradiation (≥420 nm), (**b**) The hydrogen production rate over different catalysts, (**c**) Effect pH on hydrogen evolution rate under visible light irradiation (≥420 nm), (**d**) The hydrogen production rate under different pH.

The photocatalytic performance of the catalysts was evaluated under visible light irradiation (λ ≥ 420 nm), as shown in Figure 5a,b. The hydrogen evolution rate for the Pt/Fe_3_O_4_-MSIG catalyst was 1.486 mL/h, while the corresponding rates for Pt/Fe_3_O_4_, Pt/Fe_3_O_4_-CGO, and Pt/-MSIG were only 0.105 mL/h, 0.668 mL/h, and 0.820 mL/h, respectively. These results highlight the crucial role of the catalyst support in enhancing catalytic activity. For the Pt/Fe_3_O_4_ catalysts, many defect sites on the Fe_3_O_4_ surface can negatively impact electron transfer, thereby reducing catalytic efficiency. A comparison between the Pt/Fe_3_O_4_-MSIG and Pt/Fe_3_O_4_-CGO catalysts revealed that iodine atoms play a significant role in hydrogen production. This is likely due to the presence of iodine primarily in the form of I_3_^−^ and I_5_^−^ on the graphene sheets. The *p*-orbitals of I_3_^−^ and I_5_^−^ can interact with the *p*-orbitals of graphene oxide, facilitating electron transfer. Furthermore, these iodine-containing clusters can induce spin-orbit coupling in graphene oxide, promoting spin polarization and electron inversion within the material [42].

In the context of hydrogen production, the pH of the reaction system plays a significant role in determining the catalyst’s activity, with a basic environment being essential for formaldehyde conversion. Consequently, our study focused on investigating the influence of pH on the photocatalytic performance of Pt/Fe_3_O_4_-MSIG for hydrogen generation. As shown in Figure 5c,d, the catalyst achieved its highest hydrogen production rate at pH 10, with a notable photocatalytic hydrogen evolution rate of 5.90 mL/h. Within the pH range of 7–9, triethanolamine undergoes deprotonation, leading to a decrease in the concentration of free triethanolamine in the system. However, free triethanolamine is crucial for quenching EY_3_* and facilitating charge transfer between triethanolamine and EY, both of which are negatively impacted by the reduced concentration of free triethanolamine. Conversely, excessively high pH values result in stronger electrostatic repulsion between the negatively charged catalyst surface and the deprotonated EY, which hinders the adsorption of EY dye molecules onto the catalyst surface [43].

### 2.6. Photo-Electrochemical Analysis

**Figure 6:**
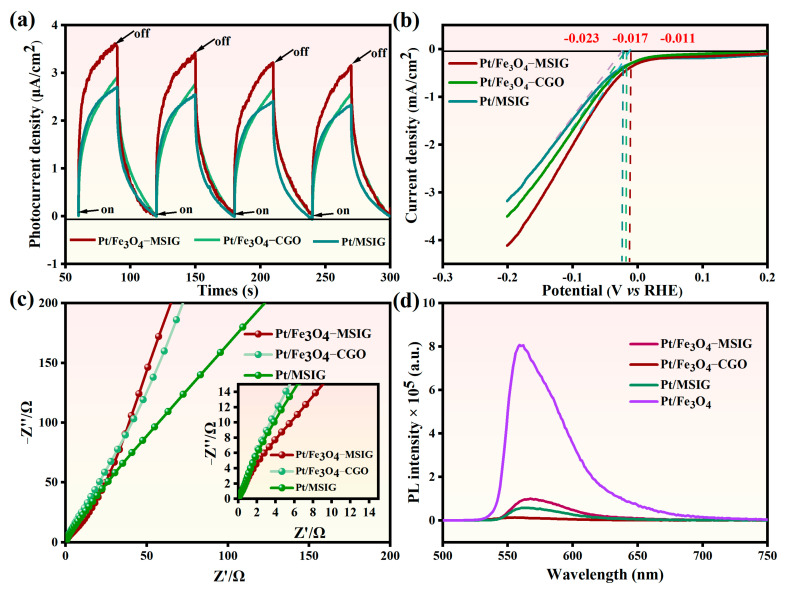
(**a**) Transient photocurrent responses, (**b**) linear-sweep voltammograms collected with a scan rate of 1 mV/s, (**c**) electrochemical impedance spectroscopy (EIS) Nyquist plots, (**d**) Photoluminescence (PL) spectra of EY-sensitized Pt/Fe_3_O_4_-CGO, Pt/Fe_3_O_4_-MSIG and Pt/MSIG catalysts.

The photo-electrochemical method and photoluminescence (PL) spectroscopy were employed to investigate the mechanism of the hydrogen evolution reaction. To facilitate a comparative analysis of charge formation and transfer across the Pt/Fe_3_O_4_-CGO, Pt/Fe_3_O_4_-MSIG, and Pt/MSIG catalysts, the same fabrication procedure was applied to prepare these catalysts as electrodes for subsequent electrochemical experiments. As shown in Figure 6a, transient photocurrent responses of Pt/Fe_3_O_4_-CGO, Pt/Fe_3_O_4_-MSIG, and Pt/MSIG electrodes, coated on ITO, were evaluated during multiple on-off cycles of intermittent irradiation (60 s). Remarkably, the Pt/Fe_3_O_4_-MSIG catalyst exhibited the highest photocurrent, suggesting a faster electron transfer rate compared to the Pt/Fe_3_O_4_-CGO and Pt/MSIG catalysts. This improvement can be attributed to the expanded graphene’s topological structure and the bridge-like configurations formed by I_3_^−^ and I_5_^−^ ions on the graphene oxide surface, which effectively shorten the electron transport path, thereby enhancing the photocurrent [44].

Hydrogen evolution overpotential and cathode current density are critical factors influencing the efficiency of hydrogen production reactions. A decrease in the hydrogen evolution overpotential and an optimized cathode current density can accelerate the hydrogen evolution reaction and enhance overall hydrogen production activity. To evaluate this, the electrochemical hydrogen generation activities of Pt/Fe_3_O_4_-CGO, Pt/Fe_3_O_4_-MSIG, and Pt/MSIG electrodes were analyzed using linear sweep voltammetry (LSV). As shown in Figure 6b, the LSV curve reveals that the Pt/Fe_3_O_4_-MSIG catalyst exhibits the highest cathode current among the tested catalysts, including Pt/Fe_3_O_4_-CGO and Pt/MSIG. This indicates that Pt/Fe_3_O_4_-MSIG favors hydrogen generation, in agreement with its high hydrogen production activity reported in silver-modified nano-structured TiO_2_ thin film [45]. Additionally, the Pt/Fe_3_O_4_-MSIG catalyst demonstrates a lower overpotential of −0.011 V compared to Pt/Fe_3_O_4_-CGO and Pt/MSIG, which significantly contributes to its superior activity.

Further insights into the charge generation and transport processes were obtained through additional electrochemical experiments. Nyquist plots derived from the electrochemical impedance spectroscopy (EIS) measurements, conducted in a 0.5 mol/L NaOH electrolyte solution, are presented in Figure 6c. A larger radius corresponds to higher charge transfer resistance. Notably, the Pt/Fe_3_O_4_-MSIG sample exhibits the smallest semicircle among the Pt/Fe_3_O_4_-CGO and Pt/MSIG electrodes, indicating the lowest resistance to electron transfer on its surface, thereby facilitating efficient electron transport. This advantageous feature can be attributed to two factors: the presence of numerous defect sites on the surface that assist in trapping metal ions and promote electron transfer between CGO and Pt nanoparticles, and the bridging structure formed by I_3_^−^ and I_5_^−^ ions on the material’s surface, which imparts a Möbius ring-like structure to graphene oxide, further enhancing electron transport on the catalyst’s surface [46].

Additional insights into the electron transport dynamics on the catalyst surface were provided by the photoluminescence (PL) spectrum analysis [47]. As shown in Figure 6d, a distinct emission peak at approximately 536 nm is observed in the PL spectrum of EY-sensitized Pt/Fe_3_O_4_. This intense emission peak indicates rapid recombination of photogenerated electrons and holes. Notably, the incorporation of Pt/Fe_3_O_4_-CGO, Pt/Fe_3_O_4_-MSIG, and Pt/MSIG catalysts results in a significant quenching of these emissions, signifying a more efficient suppression of electron-hole recombination and, consequently, improved electron transport on the catalyst surface.

**Figure 7:**
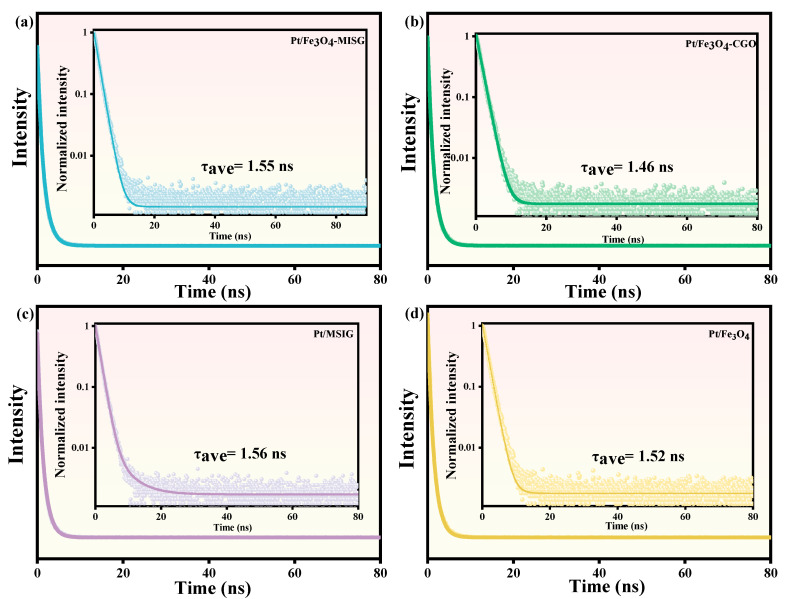
Fluorescence lifetimes of EY sensitized (**a**) Pt/Fe_3_O_4_-MSIG, (**b**) Pt/Fe_3_O_4_-CGO, (**c**) Pt/MSIG, and (**d**) Pt/Fe_3_O_4_ catalysts.

Further insights into the electron generation and transfer processes were obtained through fluorescence lifetime measurements, as shown in Figure 7. The average lifetimes of excited charge carriers for EY-sensitized Pt/Fe_3_O_4_, Pt/Fe_3_O_4_-CGO, Pt/Fe_3_O_4_-MSIG, and Pt/MSIG catalysts were 1.52, 1.46, 1.55, and 1.56 ns, respectively. The fluorescence lifetime of excited charge carriers reflects the electron transport and transfer processes on the surface of these materials. In comparison, the fluorescence lifetime of Pt/Fe_3_O_4_-MSIG is approximately twice longer than that of previously reported Pt/Fe_3_O_4_-RGO catalyst, suggesting a faster electron transfer rate on the surface of Pt/Fe_3_O_4_-MSIG. Among all the tested catalysts, Pt/Fe_3_O_4_-MSIG and Pt/MSIG exhibited the longest charge lifetimes, which indicates efficient charge separation and transfer, correlating with their highest catalytic activity for hydrogen generation reactions [48]. In contrast, Pt/Fe_3_O_4_-CGO exhibited the shortest electronic lifetime, which can be attributed to the presence of I clusters, resulting in an extended photoelectron lifetime. Considering that the triplet photoelectron lifetime in dye molecules is longer than that of singlet photoelectrons [49], the incorporation of these iodine clusters likely contributes to prolonging the lifetime of excited electrons. Additionally, a Möbius ring-like electronic transport channel is formed in the material, further enhancing the fluorescence lifetime and increasing the number of photo-generated electrons in the material [50].

### 2.7. Catalytic Mechanism

**Figure 8:**
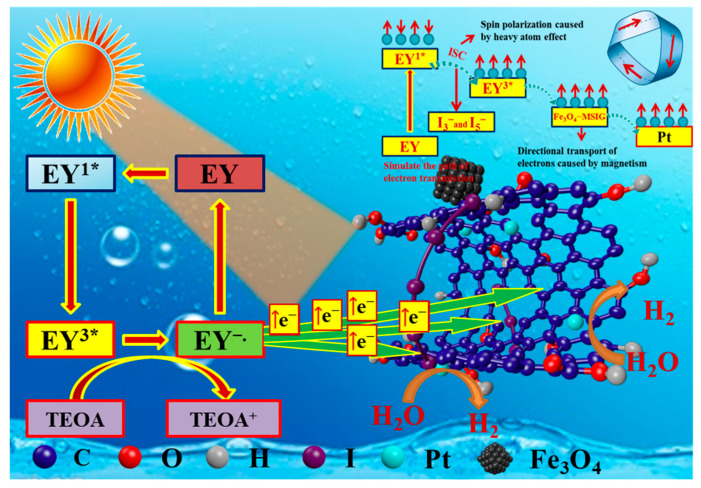
The proposed photocatalytic mechanism for hydrogen evolution from Pt/Fe_3_O_4_ MSIG catalyst under visible light irradiation.

The hydrogen production process in the dye-sensitized photolysis water system for hydrogen generation can be summarized as follows (Figure 8): Dye molecules are first adsorbed onto the surface of the catalyst. Upon adsorption, these dye molecules absorb photons under visible light irradiation, leading to the formation of singly excited dye molecules (EY_1_*). These excited states are unstable and undergo intersystem crossing to form a more stable triply excited state (EY_3_*). The triply excited dye molecules are quenched by triethanolamine, generating the EY-species, which in turn produce electrons. These generated electrons are transferred to the surface of Pt through the high conductivity of graphene. The H^⁺^ ions adsorbed on the Pt surface gain electrons and are reduced to hydrogen gas.

**Figure 9:**
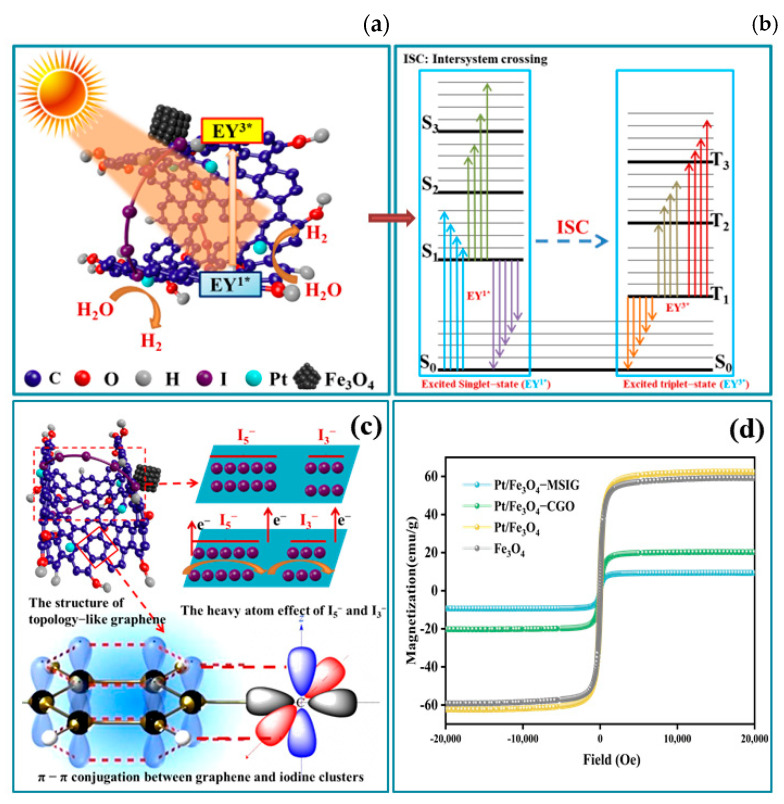
(**a**,**b**) The transition between the singlet and triplet states of the dye molecule eosin; (**c**) The interaction between Cluster I_3_^−^ and I_5_^−^ and graphene oxide and its heavy atom effect; (**d**) hysteresis loops of different catalysts.

In this process, the transformation between the singlet and triplet states of the dye molecules plays a crucial role. As shown in Figure 9a,b, the heavy-atom effect of I_3_^−^ and I_5_^−^ clusters on the graphene surface can partially overcome the spin-forbidden transition, enhancing the singlet-to-triplet transition of dye molecules and promoting the population of excited electrons in the triplet state. Since triplet electrons have parallel spins, the increased population of triplet excited states in the dye facilitates electron spin polarization, which, in turn, enhances hydrogen production. This suggests that the spin property of the electrons plays a pivotal role in electron transport and contributes to the overall efficiency of hydrogen generation [50]. Furthermore, as shown in Figure 9c, the *p*-orbitals of I_3_^−^ and I_5_^−^ can conjugate with the *p*-orbitals of graphene oxide, thereby promoting electron transfer between the catalyst and the dye. This synergy between the iodine clusters and the graphene oxide further enhances the photocatalytic performance of the system.

A critical observation in this research was the close correlation between the catalytic activity of the three magnetic catalysts and their intrinsic magnetic properties (Figure 9d). Among Fe_3_O_4_ (Ms = 58.18 emu/g), Pt/Fe_3_O_4_ (Ms = 62.05 emu/g), Pt/Fe_3_O_4_-CGO (19.84 emu/g), and Pt/Fe_3_O_4_-MSIG (Ms = 9.18 emu/g), Pt/Fe_3_O_4_-MSIG exhibited the highest hydrogen evolution activity during the first round of hydrogen production, indicating that catalysts with lower saturation magnetization (Ms) tended to display higher catalytic activity.

To explain the underlying mechanism, we considered the role of the external magnetic field. Under such a field, electrons are deflected due to the Lorentz force, which gives rise to the Hall effect within the material. The Hall resistance is highly sensitive to changes in the external magnetic field, and variations in this resistance suggest that, under the influence of the external magnetic field, the deflection and transmission resistance of photogenerated electrons in the MSIG layer differ. Catalysts with higher intrinsic magnetic properties experience increased deflection and higher transmission resistance for photogenerated electrons due to the magnetic field, thereby inhibiting the transfer and transport of these charges.

Among the catalysts, Pt/Fe_3_O_4_, which has a relatively high saturation magnetization, exhibited the greatest deflection and transmission resistance of photogenerated electrons, followed by Pt/Fe_3_O_4_-CGO. Pt/Fe_3_O_4_-MSIG, which has the lowest saturation magnetization, demonstrated the smallest electron deflection and transmission resistance. This observation is consistent with the results of the photoelectrochemical characterization, reinforcing the idea that the intrinsic magnetic properties of the catalysts play a crucial role in modulating their electron transfer capabilities and, consequently, their catalytic activity [51].

**Figure 10:**
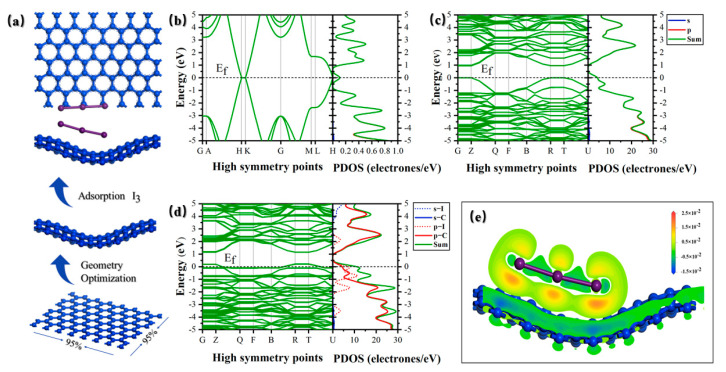
(**a**) Schematic diagram of curved graphene and MSIG construction. Band structures and Projected density of states (PDOS) of (**b**) planar graphene (**c**) curved graphene and (**d**) MSIG. (**e**) Charge transfer (units of electron·bohr^−3^) along the chain for I_3_^−^, shown on the YZ plane.

To validate our experimental findings, we investigated the bandgap structure and electron transport pathways of the material through DFT theoretical calculations (Figure 10). In comparison to pristine 2D planar graphene, the bent graphene structure introduces a bandgap of approximately 0.9 eV [52]. This bandgap, induced by the bending effect, reduces the recombination probability of photogenerated carriers and extends the average carrier lifetime. Simultaneously, iodine adsorptive doping shifts the Fermi energy level of bent graphene downward, conferring P-type semiconductor characteristics [53,54,55]. The differential charge density plot reveals that the I_3_^−^ chains gain charge in the π* orbitals located in a plane perpendicular to the graphene sheet, thereby facilitating an electron transfer pathway [56].

## 3. Materials and Methods

Details of the material preparation methods and DFT calculations are given in Supplementary Materials [57,58,59,60,61,62,63,64,65,66].

## 4. Conclusions

In this study, we developed a Möbius strip-like electron transport channel through a simple and cost-effective experimental approach by modifying CGO material with I_3_^−^ and I_5_^−^. The sensitized Pt/Fe_3_O_4_-MSIG catalyst thus prepared exhibited the highest activity for the hydrogen evolution reaction. The superior catalytic performance can be attributed to several key factors. First, under an external magnetic field, electrons experience deflection due to the Lorentz force, and the Hall effect within the material influences its Hall resistance, thereby affecting its conductivity. Second, the incorporation of I_3_^⁻^ and I_5_^⁻^ significantly enhances electron spin-flip processes, greatly expanding electron transmission pathways within the material. This leads to the formation of a Möbius strip-like electron transport path, improving the utilization efficiency of photoelectrons, markedly extending the fluorescence lifetime, and ultimately boosting the catalyst’s hydrogen production performance. The conclusions are strongly supported by results from photo-electrochemical analysis and photoluminescence (PL) spectra. Our findings offer theoretical guidance for the design of high-efficiency carbon materials aimed at shortening electron transport paths and promoting efficient electron transfer.

## Data Availability

Data will be made available on request.

## Supplementary Materials

The following supporting information can be downloaded at: https://www.mdpi.com/article/10.3390/molecules30061302/s1, Material preparation methods and DFT calculations.
