# Supplementary material for: Boosting Visible-Light-Driven Hydrogen Evolution Enabled by Iodine-Linked Magnetically Curved Graphene with Mobius-like Electronic Paths
Source: Molecules. 2025 Mar 13;30(6):1302. doi: 10.3390/molecules30061302 (PMC11946169; doi:10.3390/molecules30061302)
Supplement: Supplementary file 1 [file molecules-30-01302-s001.zip › molecules-3487363-supplementary.pdf]

Supporting information

Boosting visible-light-driven hydrogen evolution enabled by Iodine-linked magnetically curved graphene with Mobius-like electronic paths

Liangjun Cai[1], Hongxia Liu[1,*], Xiaoxiao Yan[2,*]

[1]Jiangxi Province Key Laboratory of Environmental Pollution Prevention and Control in Mining and Metallurgy, Jiangxi University of Science and Technology, Ganzhou 341000, P.R. China

[2] Key Laboratory for Green Chemistry of Jiangxi Province, Jiangxi Normal University, Nanchang 330022, P.R. China

*Corresponding author.

E-mail address: liuhongxiajxust@163.com (Hongxia Liu)

3.Experimental

3.1. Materials

All chemicals utilized in this experiment were procured from commercial sources and were directly employed without any additional purification steps. This includes graphite powder from Sinopharm Chemical Reagent Co., Ltd. (CP grade), phosphorus pentoxide (P_2O_5) from Kelong Chemical Reagent Factory (AR grade), sulfuric acid (H_2SO_4) from Liangyou Reagent Co., Ltd. (AR grade), sodium persulfate ($K_2S_2O_8$) from Xilong Chemical Industry Co., Ltd. (AR grade), potassium permanganate ($KMnO_4$) from J&K Scientific Ltd. (AR grade), hydrogen peroxide (H_2O_2) from Hongyan Chemical Reagent Factory (AR grade), sodium nitrate ($NaNO_3$) from J&K

Scientific Ltd. (AR grade), ethylenediamine ($C_2H_8N_2$) from Xilong Chemical Co., Ltd. (AR grade), sodium borohydride ($NaBH_4$) from Sinopharm Chemical Reagent Co., Ltd. (AR grade), hydrochloric acid (HCl) from Liangyou Reagent Co., Ltd. (AR grade), palladium chloride ($PdCl_2$) from Tianjin Kemiou Chemical Reagent Co., Ltd. (AR grade), Ferric Chloride Hexahydrate ($FeCl_3 \cdot 6H_2O$) from Xilong Chemical Co., Ltd. (AR grade), Hydroiodic acid (HI) from Shanghai Macklin Biochemical Technology Co., Ltd. (AR grade), Triethanolamine ($C_6H_{15}NO_3$) and sodium hydroxide (NaOH), both from Xilong Chemical Co., Ltd. (AR grade), Polyvinylpyrrolidone (PVP) from Xilong Chemical Co., Ltd. (AR grade) and formaldehyde (HCHO) also from Xilong Chemical Co., Ltd. (AR grade).

3.2. Catalyst preparation

3.2.1 Preparation of the CGO

Graphene oxide (GO) was synthesized utilizing a modified Hummers method. To obtain reduced graphene oxide (RGO) dispersions in water at a concentration of 0.66 $mg \cdot mL^{-1}$, the GO was then reduced with sodium borohydride ($NaBH_4$) serving as the reducing agent.[57, 58]. Crumpled graphene oxide is produced through a high-temperature heat treatment process involving graphene oxide. Here's the detailed experimental protocol: Initially, the graphene oxide is fragmented into smaller pieces and positioned inside a tubular furnace. The furnace is then heated to 250°C at a rate of 10°C per minute. Once the desired temperature is reached, the material is maintained

at 250°C for a duration of 5 minutes. Following this, the furnace is cooled down to 80°C, all under a nitrogen (N_2) atmosphere.[59]

3.2.2 Preparation of the MSIG

As shown in Fig.1, weigh 100 mg of CGO and 70 mg of iodine, add 35 mL of deionized water, and use ultrasonic treatment (40 kHz, 2000 W) until the solid material is uniformly dispersed in the water. Under continuous stirring, slowly add 10 mL of hydroiodic acid (47 wt%) to the resulting purple-black solution. Then, transfer the mixed solution to a Teflon-lined autoclave for hydrothermal reaction (200°C, 20 h). After the iodination process, allow the autoclave to cool down to room temperature. Next, separate the product from the suspension through suction filtration using an organic filter membrane (0.22 μm) and repeatedly wash it with ethanol and deionized water to remove any unreacted hydroiodic acid and iodine. Finally, freeze-dry the sample to obtain Möbius-strip-like iodinated graphene (MSIG) with a topology-like structure.

3.2.3 Preparation of the Fe_3O_4 and Pt/ Fe_3O_4-MSIG

The monodisperse Fe_3O_4 microspheres were synthesized using the hydrothermal method. Specifically, 0.54 g of iron(III) chloride hexahydrate, 1.2 g of sodium citrate, and 0.2 g of urea were dissolved in 40 mL of deionized water. Then, under continuous stirring, 0.2 g of sodium polyacrylate was slowly added until the solid substances were completely dissolved. Subsequently, the solution was transferred to a 50 mL Teflon-lined autoclave, sealed, and maintained at 200°C for 12 hours. The resulting product

was separated by magnetic separation, repeatedly washed with deionized water and absolute ethanol, and finally freeze-dried to obtain monodisperse Fe_3O_4 microspheres. 90 mg of Fe_3O_4 and 0.2 g PVP were redispersed in 40 mL of deionized water using ultrasonication. Under vigorous stirring, 1.2 g of sodium citrate was dissolved into the prepared solution. Then, 2.5 ml of H_2PtCl_6 solution and 100 mg of iodinated graphene were added to the solution. Following that, the mixed solution was transferred to a 50 mL Teflon-lined hydrothermal autoclave, sealed, and reacted at 200°C for 12 hours. After the reaction, the product was obtained through magnetic separation and washed repeatedly with deionized water and anhydrous ethanol. Finally, the sample was freeze-dried to obtain the final product, Pt/ Fe_3O_4-MSIG.

3.3. Photocatalytic activities measurement

Photocatalytic experiments were performed at room temperature in a sealed Pyrex flask (180 mL) with a flat window and a silicone rubber septum for sampling. 2.2 mg catalysts and 70 mg Eosin Y (EY) were added to triethanolamine solution in the reactor. The Xenon lamp (HSX-UV 300, NBeT) with a 420 nm cut-off filter was used as a light source to trigger the photocatalytic reaction. The amount of hydrogen evolution was measured using gas chromatograph (Agilent 6820, TCD, 13 X columns, Ar carrier). A continuous magnetic stirrer was applied in order to keep the catalyst in suspension status during the experiment.

3.4. DFT experiments

The electronic properties of the materials are investigated based on density-functional theory (DFT) [60, 61]. The Cambridge Sequential Total Energy Package (CASTEP) in Materials Studio 2023 is used and combined with a projection augmented wave (PAW) method based on a generalized gradient approximation (GGA) of the Perdew–Burke–Ernzerhof (PBE) exchange correlation function for structure optimization and acquisition of electronic properties [62-66]. The integration in the Brillouin zone is based on the Monkhorst-Pack scheme using a 2 × 2 × 1 k-point lattice centered on the Gamma points for all calculations. The truncation energy of the plane-wave basis functions is taken to be 400 eV; The energy convergence criterion is 1 × 10-5eV/atom, the maximum allowable force is 0.03 eV $Å^{-1}$, the maximum atomic displacement convergence criterion is 10^{-3} Å, and the in-lattice pressure convergence criterion is 0.05 GPa. The DFT-D3 method in the Grimme scheme was used to describe the van der Waals (vdW) interactions. As Fig. 1. (a) shows, a$\begin{pmatrix} 3 & -3 \\ 7 & -7 \end{pmatrix}$supercell of planar armchair graphene is first constructed (with dimensions of approximately 17.22*12.78 Å in length and width). Subsequently, the lattice constants of a and b were reduced to 95% of the original ones, and a 15 Å vacuum layer was added in the c direction and geometrically optimized to obtain a wavy graphene, and then the I_3 chains were attached in the form of adsorption on the surface of curved graphene, to give our iodinated graphene model (MSIG).

3.5. Characterizations

The Tecnai-G2-F30 field emission transmission electron microscope, operating at a 300 kV accelerating voltage, was utilized for conducting Transmission Electron Microscopy (TEM) and High-Resolution Transmission Electron Microscopy (HRTEM) analyses. Additionally, the X-ray diffraction (XRD) patterns of the prepared catalysts were acquired using a Rigaku B/Max-RB X-ray diffractometer, which employed nickel-filtrated Cu Kα radiation operated at 40 kV and 40 mA. Furthermore, the X-ray Photoelectron Spectroscopy (XPS) measurements were carried out on a VG Scientific ESCALAB 210-XPS photoelectron spectrometer, utilizing a Mg Kα X-ray source.

3.6. Electrochemical performance

The electrochemical measurements were conducted using an electrochemical analyzer (CHI660E) in a custom-built, three-electrode quartz cell. This cell comprised an organic glass enclosure featuring a quartz window and a 1.2 cm diameter opening opposite to the work electrode. The work electrode was fabricated by directly drop-coating a sample suspension onto a pre-cleaned indium tin oxide glass (ITO) surface. A Pt plate served as the counter electrode, while Hg-HgO as the reference electrode. The electrolyte consisted of a 0.5 M aqueous NaOH solution, devoid of any additives. The source of visible light irradiation was a 300 W Xe arc lamp system, accompanied by a 420 nm cut-off filter.

References

57. Liu, H.; Wang, M.; Ma, J.; Lu, G., Hydrogen generation from toxic formaldehyde catalyzed by low-cost Pd–Sn alloys driven by visible light. *J. Mater. Chem. A* **2020**, 8, (19), 9616-9628.

58. Liu, H.; Wang, M.; Ma, J.; Lu, G., Modulation of HCHO, H_2O and H adsorption on AgPd cocatalyst by optimizing of selective exposed facet to enhancing the efficiency of conversion toxic formaldehyde into hydrogen driven by visible light. *J. Catal.* **2019,** 375, 493-506.
59. Zhang, W.; Li, Y.; Peng, S., Facile Synthesis of Graphene Sponge from Graphene Oxide for Efficient Dye-Sensitized H_2 Evolution. *ACS Appl Mater Interfaces* **2016,** 8, (24), 15187-95.
60. Hohenberg, P.; Kohn, W., Inhomogeneous Electron Gas. *Physical Review* **1964,** 136, (3B), B864-B871.
61. Kohn, W.; Sham, L. J., Self-Consistent Equations Including Exchange and Correlation Effects. *Physical Review* **1965,** 140, (4A), A1133-A1138.
62. Clark, S.; Segall, M.; Pickard, C.; Hasnip, P.; Probert, M.; Refson, K.; Payne, M., First principles methods using CASTEP. *Z. Kristallogr.* **2005,** 220.
63. Blöchl, P. E., Projector augmented-wave method. *Physical Review B* **1994,** 50, (24), 17953-17979.
64. Perdew, J. P.; Burke, K.; Ernzerhof, M., Generalized Gradient Approximation Made Simple. *Phys. Rev. Lett.* **1997,** 78, (7), 1396-1396.
65. Perdew, J. P.; Burke, K.; Ernzerhof, M., Perdew, Burke, and Ernzerhof Reply. *Phys. Rev. Lett.* **1998,** 80, (4), 891-891.
66. Kresse, G.; Joubert, D., From ultrasoft pseudopotentials to the projector augmented-wave method. *Physical Review B* **1999,** 59, (3), 1758-1775.
